# Targeting EGFR in Esophagogastric Cancer

**DOI:** 10.3389/fonc.2020.553876

**Published:** 2020-12-08

**Authors:** Steven B. Maron, James Xu, Yelena Y. Janjigian

**Affiliations:** ^1^ Gastrointestinal Oncology Service, Division of Solid Tumor Oncology, Department of Medicine, Memorial Sloan Kettering Cancer Center, New York, NY, United States; ^2^ Computer Engineering Program, Columbia University, New York, NY, United States

**Keywords:** esophageal cancer, gastric cancer, gastroesophageal cancer, epidermal growth factor receptor, cetuximab, panitumumab, targeted therapy, ctDNA

## Abstract

Esophagogastric cancer (EGC) remains a major cause of cancer-related mortality. Overall survival in the metastatic setting remains poor, with few molecular targeted approaches having been successfully incorporated into routine care to-date: only first line anti-HER2 therapy in ERBB2-expressing tumors, second line anti-VEGFR2 therapy with ramucirumab in unselected patients, and pembrolizumab in PD-L1 expressing or MSI-H patients. EGFR inhibitors were extensively studied in EGC, including phase III trials with cetuximab (EXPAND), panitumumab (REAL3), and gefitinib (COG). All three trials were conducted in unselected populations, and therefore, failed to demonstrate clinical benefit. Here, we review previous attempts at targeting EGFR in EGC and potential future biomarkers for targeting this pathway in patients with *EGFR*-amplified tumors.

## Introduction

Esophagogastric cancer (EGC), consisting of esophagogastric junction adenocarcinoma (EGJ) and distal gastric adenocarcinoma (GC), remains a leading cause of cancer-related mortality (1). In the metastatic setting, median overall survival remains approximately 11 months with optimal palliative chemotherapy in epidermal growth factor receptor 2 (*ERRB2*) negative patients (2). Molecularly, EGC consists of four distinct subtypes: Epstein Barr Virus-positive (EBV+), microsatellite instability-high (MSI-H), chromosomally unstable (CIN), and genomically stable (GS) (3, 4). While EBV+ and MSI-H tumors have frequent responses to PD-1 inhibition, these represent only ~10–15% of metastatic EGC patients; the vast majority of patients have CIN tumors. CIN tumors characteristically acquire chromosomal instability earlier in their tumorigenesis, which results in copy number amplification of numerous receptor tyrosine kinases, including *ERBB2*, *EGFR*, *MET*, *KRAS*, and *FGFR2* (5–8). Clinical trials of agents targeting these pathways have had mixed results in EGC. However, interpretation of these results requires understanding both the agents used and the study population. 

**Figure 1:**
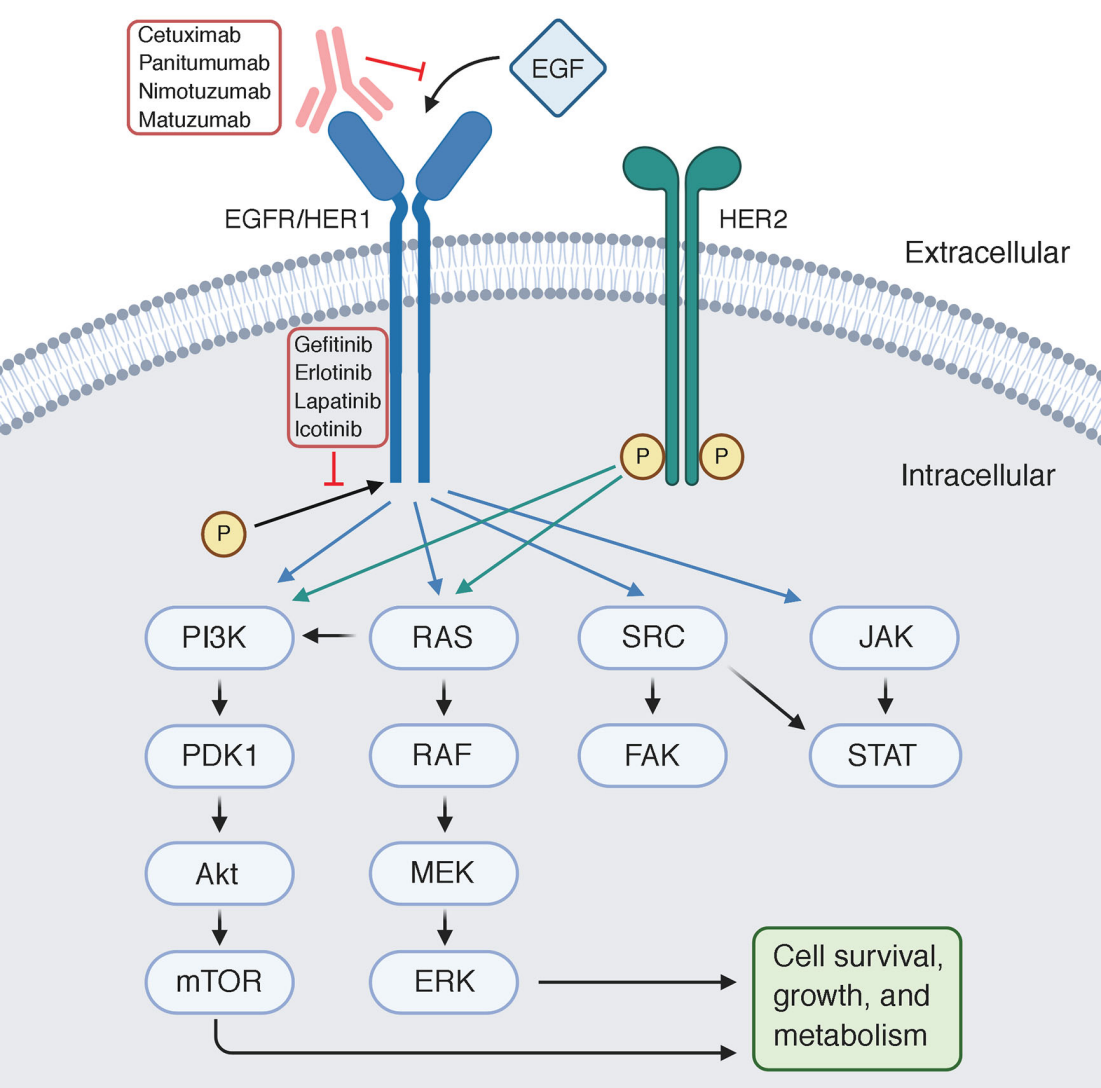
Schematic of EGFR and its downstream pathways with monoclonal antibodies exerting their effects at the extracellular domain, and small molecule inhibitors inhibiting phosphorylation intracellularly.

Epidermal growth factor receptor (EGFR or ERBB1) is a 170-kDa transmembrane receptor. While other ERBB family members such as ERBB2 and ERBB3 depend on heterodimer complexes to generate downstream signaling, EGFR binds to multiple ligands, including EGF, which results in homo- and hetero-dimer formation, and subsequent tyrosine phosphorylation of the cytoplasmic domain. Ultimately, EGFR activation triggers a signaling cascade of cell proliferation and survival signaling *via* activation of MAPK, STAT5, and Ras-Raf-MEK pathways (**Figure 1**) (9, 10). EGFR-overexpressing EGC tumors are associated with higher stage, more poorly differentiated histology, increased vascular invasion, and potentially shorter survival (11, 12).

EGFR is highly expressed in many cancers and amplified in 8.5% of solid tumors, including colorectal cancer (16.3%), non-small cell lung cancer (9%), genitourinary cancers (8.1%), and breast cancer (7.3%) (13). EGFR is also expressed on approximately 30% of EGC tumors and amplified in 6% of patients with metastatic EGC (14–16). Therefore, EGFR-targeting agents entered the clinic in multiple cancer types, with mixed success.

## EGFR Therapy Patient Selection

Gefitinib, a small molecule inhibitor of EGFR, entered phase I trials in 1998 and demonstrated a 25% ORR in unselected patients with NSCLC. During phase II trials, responses were more likely in patients with lung adenocarcinoma who were female, never-smokers, and of Asian origin; and it was not until 6 years later that *EGFR* mutations were published as biomarkers of response to EGFR inhibitors (17). Applying this knowledge, the phase III I-PASS trial selected East Asian patients with lung adenocarcinoma and minimal smoking history and demonstrated that only *EGFR*-mutated patients benefited from gefitinib (18). In the past decade, this finding has led to approval of gefitinib, erlotinib, afatinib, osimertinib, dacomitinib, and necitumumab in the 15% of patients with *EGFR*-mutated NSCLC. EGFR inhibition with cetuximab in colorectal cancer underwent a concurrent transformation from benefit across the patient population (19), to those without *KRAS* mutations (20, 21), and now even more limited to those with left-sided pan-*RAS* wildtype tumors (22, 23). In both lung and colon adenocarcinomas, efficacy is dependent on patient selection.

## Targeting EGFR in Metastatic EGC (mEGC)

Numerous EGFR-targeting phase II studies evaluated cetuximab (24–37), panitumumab (38, 39), nimotuzumab (40, 41), lapatinib (42, 43), erlotinib (44, 45), gefitinib (46), matuzumab (47), and icotinib (48) in mEGC patients (**Table 1**). As a monotherapy in an unselected population, cetuximab had modest benefit with an ORR of 5% and a median progression-free survival (mPFS) of 4.0 months in these patients—including one patient with an 11.3 month PFS—which suggested that a small subset of patients benefits from EGFR-directed therapy (36). Subsequent evaluation with gefitinib in EGFR-expressing (46), and erlotinib+radiation or icotinib in *EGFR*-amplified patients (45, 48) further supported this premise.

**Table 1:** Phase II mEGC trials evaluating EGFR inhibitors evaluating predictive and/or prognostic biomarkers.

Author Year (Citation)	Population	N	Treatment	mPFS mo. (95% CI)	ORR (DCR; 95% CI)	mOS mo.(95% CI)	EGFR Expression/Amplification	Additional Biomarkers Evaluated
Moehler et al., 2011 (49)	1L GEJ/GC	49	FOLFIRI /Cetuximab	9.0 (7.1–15.6)	46% (79%; 31–61%)	16.5 (11.7–30.1)	IHC 3+ correlated with ORR, not PFS/OS	PTEN expression correlated with improved ORR and mOS, no response correlation for *KRAS* mutations
Liu et al., 2017 (28)	2L GC	61	FOLFIRI/Cetuximab	4.6 (3.6–5.6)	33% (83%; 20.7–45.9%)	8.6 (7.3–9.9)	No ORR, PFS, or OS correlation (Cutoff 10% EGFR+)	High baseline VEGF correlated with ORR, PFS, and OS
Pinto et al., 2007 (FOLCETUX) (27)	1L GEJ/GC	38	FOLFIRI /Cetuximab	8.0 (7.0–9.0)	44.1% (91.2%; 27.5–60.9%)	16 (9.0–23.0)	No ORR correlation (Cutoff 50% EGFR+)	–
Lordick et al., 2010 (26) / Luber et al., 2011 (29)	1L GC	52	FUFOX /Cetuximab	7.6 (4.4–10.4)	62% (81%; 46–76%)	9.6 (6.6–12.6)	No expression ORR, PFS, OS correlation (Cutoff 1% of cells). EGFR copy number >4 and CEP7 aneusomy correlated with increased OS and TTP	pEGFR expression correlated with worse ORR and TTP; KRAS exon 12/13 mutations only
Han et al.,2009 (30)	1L GEJ/GC	40	FOLFOX6 / Cetuximab	5.5 (4.5–6.5)	47.5% (87.5%, 32.0–63.0%)	9.9 (NA)	EGFR expression correlated with multivariate TTP	EGFR expression with low serum EGF and TGF-beta correlated with multivariate TTP and OS
Gold et al., 2010 (36)	2L E/GEJ Adeno	55	Cetuximab	1.8 (1.7–1.9)	5% (16%; 1–16%)	4.0 (3.2–5.9)	–	No ORR or OS correlation for EGFR mutations, EGF, or VEGF expression
Du et al., 2015 (40)	1L advanced GEJ/GC Adeno	62	Cisplatin/S-1	7.2	58.1 (90.3%)	14.3	No PFS/OS benefit. Possible PFS detriment in gastric cancer (cutoff: 2/3+ IHC)	–
			Cisplatin/S-1 /Nimotuzumab	4.8	54.8 (90.3%)	10.2		
Satoh et al., 2015 (41)	2L+ GC	83	Irinotecan	2.8 (1.2–3.1)	0% (46.2%)	7.6 (4.9–10.5)	Non-significant trend towards ORR/PFS/OS benefit (cutoff:2/3+ IHC)	–
			Irinotecan/ Nimotuzumab	2.4 (1.8–3.7)	33.3% (47.4%)	8.2 (5.6–10.1)		
Rao et al., 2010 (47)	1L advanced GEA, EGFR IHC	72	ECX	7.1 (4.4–8.5)	58% (75%, 41–74%)	12.2 (9.8–13.8)	(cutoff: only patients with membranous EGFR staining enrolled)	–
			ECX/Matuzumab	4.8 (2.9–8.1)	31% (60%; 17–49%)	9.4 (7.5–16.2)		
Huang et al., 2016 (48)	2L E/GEJ SCC IHC 3+ or FISH amp	54	Icotinib	1.7 (1.3–3.1)	16.7% (46.3%; 7.2–28.1%)	5.0 (4.6–7.2)	Informal trend towards improved ORR, PFS, and OS, in EGFR IHC 3+ or FISH+ (4+ copies or EGFR:Chr7>2 enrolled)	
Labonte et al., 2016 (43)	1L GEJ/GC	67	Capecitabine/Lapatinib	3.3 (2.9–4.3)	17.9% (64.2%; 9.6–29.2%)	6.3 (5.0–9.1)	–	No ORR, PFS, OS correlation for SNPs in *EGF*, *EGFR*, *HER2*, *VEGF*. *HER3* mRNA expression correlated with ORR
Moehler et al., 2018 (EORTC 40071) (49)	1L GC	28	ECF/X + Placebo	8	21%	13.8	Non-significant trend towards ORR and PFS benefit in 2/3+ IHC or FISH+	–
			ECF/X + Lapatinib	5.9	43%	10.1		
Wainberg et al., 2011 (44)	1L E/GEJ Adeno	38	FOLFOX /Erlotinib	5.5 (3.0–7.4)	45% (84%; 30.0–61.0%)	11 (8.0–17.4)	No response correlation in *EGFR* FISH ratio >2)	No response correlation for ERBB2 amplification, HER3 over-expression, or mutations in *EGFR, KRAS, BRAF, PI3K*
Iyer et al., 2013 (45)	E/GEJ Adeno or SCC stage I-IV	17	Erlotinib/ Radiation Therapy	4.5 (2.4–7.3)	NA	7.3 (3.8–22.3)	No expression response correlation (cutoff: membranous EGFR staining) Non-significant trend towards PFS and OS benefit in *EGFR* FISH+ and polysomy)	No response correlation with p-EGFR expression
Janmaat et al., 2006 (46)	E SCC or Adeno	36	Gefitinib	1.9 (1.6–2.6)	2.8% (30.6%; 0.1–14.5%)	5.4 (0–11.0)	Non-significant trend towards PFS benefit in IHC 3+	Low p-Akt expression correlated with improved PFS, *KRAS* mutation correlated with lower PFS, no response correlation for p-ERK status

Countless combinations of EGFR inhibitors with chemotherapy demonstrated mixed results. Whereas some demonstrated mPFS exceeding 8 months (48, 49), others suggested that adding EGFR inhibitors may even be detrimental (40). Biomarker analyses sought to delineate a sub-population that benefited from EGFR inhibition, but utilized inadequate and inconsistent expression and copy number definitions. In biomarker analyses, EGFR expression demonstrated mixed results. Many studies defined low EGFR expression cutoffs, and therefore failed to identify a correlation with response and survival (25, 26, 28, 40, 45, 47), which mirrors the lack of trastuzumab benefit in ERBB2 low-expressors in ToGA (50). However, response and survival benefits were suggested in nearly all studies that used more clinically relevant cutoffs of IHC 2/3+ (30, 41, 42, 46, 48, 49) or gene amplification (29, 42, 45, 48) (**Table 1**). One study indicated that VEGF expression may be prognostic for response to therapy (28), as might EGFR expression in conjunction with *EGFR* amplification or low EGF expression (30). Patients with gastroesophageal junction tumors demonstrating increased *EGFR* gene copy number or expression may also derive more benefit than those with gastric primary tumors (29, 40). These findings are all limited by small sample size, inconsistent definitions of EGFR-expression and amplification, tumor heterogeneity, and patient population.

In colorectal cancer, absence of RAS mutations and sidedness were found to be better predictors of response than EGFR expression or amplification (21, 51). However, activating mutations in *RAS* and *BRAF* are less commonly found in EGC, and so no correlation was seen between *KRAS*, *BRAF*, or *PIK3CA* mutations and survival in mEGC trials (26, 49). All of these correlative biomarkers were evaluated in small phase II studies—mostly single arm in unselected patients—and utilized inconsistent expression and copy number cutoffs. Consequently, these studies were insufficiently powered to identify a predictive biomarker. Therefore, subsequent phase III trials proceeded in unselected populations.

Based upon these findings, first-line phase III trials of chemotherapy in combination with cetuximab (EXPAND) and panitumumab (REAL3) were conducted in patients with esophagogastric cancer (52, 53) (**Table 2**). In EXPAND, 904 first-line advanced EGC patients were randomized to receive 3-week cycles of twice daily capecitabine 1,000 mg/m^2^ on days 1–14 and IV cisplatin 80 mg/m^2^ with or without cetuximab 400 mg/m^2^ on day 1 followed by 250 mg/m2 weekly with a PFS primary endpoint. Addition of cetuximab failed to improve mPFS (4.4 months with cetuximab *vs* 5.6 months without; HR 1.09; 95% CI 0.92–1.29; p = 0.32) and median overall survival (mOS) (9.4 months with cetuximab *vs* 10.7 months without; HR 1.00; 95% CI 0.87–1.17; p = 0.95). EGFR IHC was evaluated as a predictive biomarker, and most patients exhibited little to no staining. However, amongst patients with the top 6% of EGFR expression, there was a trend towards improved mPFS (HR 0.62; 95% CI 0.28–1.35) and mOS (HR 0.68; 95% CI 0.34–1.39), whereas no beneficial trend was seen in patients with less EGFR expression (54). These findings suggest that there may be a select population that benefits from cetuximab, though too small to be effectively studied in an unselected population. A similar issue was seen in REAL3.

**Table 2:** Phase III mEGC trials evaluating EGFR inhibitors including subset analysis by EGFR expression or *EGFR* amplification.

Trial	N	Treatment	mOS(mo)	HR	mPFS(mo)	HR	ORR	Stratification	mPFS(mo)	HR (95% CI)	mOS(mo)	HR (95% CI)
**Lordick et al. (** 52, 54 **)** EXPAND	904	1L Cis/5FU + Placebo	10.7	1	5.6	1.09 p = 0.32	29%	EGFR IHC > 220 (top 3%)	4.1	0.29 (0.09–0.96)	8.6	0.39 (0.12–1.25)
		1L Cis/5FU + Cetuximab	9.4		4.4		30%		7.5		19.9	
**Waddell et al. (** 53 **)** REAL3	553	1L Epi/Oxali/Cape + Placebo	11.3	1.37 p = 0.013	7.4	1.22	42%	EGFR FISH (EGFR:CNTNAP2 >5)	4.57	2.19 (0.8–6.01)	10.53	1.26 (0.46–3.44)
		1L Epi/Oxali/Cape + Panitumumab	8.8		6		46%		2.3	1.45 (0.67–3.13)	5.69	1.57 (0.72–3.38)
**Dutton et al., Petty et al. (** 55, 56 **)** COG/ TRANS-COG	450	2L Placebo	3.67	0.9	1.17	0.8	~1%	EGFR FISH amplification*	0.97	0.29 (0.10–0.83) p = 0.021	1.7	0.21 (0.07–0.64) p = 0.006
		2L Gefitinib	3.73		1.57		~4%		1.87		4.17	

*EGFR amplification according to previously published criteria (57). Per prior criteria in 340 patients in TRANS-COG subset of COG.

mPFS, Median progression-free survival; mo, months; ORR, Objective response rate; DCR, Disease control rate; mOS, Median overall survival.

FOLFIRI, folinic acid, fluorouracil and irinotecan; FOLFOX6/FUFOX, oxaliplatin, leucovorin, fluorouracil, S-1 - tegafur/gimeracil/oteracil; ECF/X, epirubicin, cisplatin, 5-fluorouracil/capecitabine.

In REAL3, 553 untreated advanced EGC patients were randomized to receive day 1 IV epirubicin 50 mg/m^2^, day 1 IV oxaliplatin 130 mg/m^2^, and daily capecitabine 1,250 mg/m^2^ (ECX) with or without panitumumab 9 mg/kg on day 1 of a 3-week cycle with a primary endpoint of OS (53). Accrual was terminated after interim analysis revealed that patients who received ECX and panitumumab had a significantly shorter mOS of 8.8 *vs* 11.3 months (HR 1.37, 95% CI 1.07–1.76, p = 0.013) than patients who did not receive panitumumab (53). Patients with *EGFR*-amplified tumors trended non-significantly towards having inferior progression-free and overall survival, regardless of treatment arm, suggesting that *EGFR* amplification portends a worse prognosis. Even in patients with tissue or plasma ddPCR *EGFR:CNTNAP2* copy number ratio >2 (6.2%) or >5 (2.7%), the addition of panitumumab failed to prolong progression-free and overall survival (58). One explanation is that these disappointing results reflect an interaction between panitumumab and an epirubicin-containing chemotherapy regimen, as all phase 2 and 3 mEG trials adding EGFR inhibition to an anthracycline triplet trended towards inferior survival (38, 42, 47, 53). In fact, in its initial form, the REAL3 regimen caused unacceptable toxicity, and therefore required an unplanned formal dose-finding study leading to a modified regimen in which oxaliplatin was reduced from 130 mg/m2 to 100 mg/m^2^ and capecitabine from 1,250 mg/m^2^ to 1,000 mg/m^2^/d. However, reduced dose-intensity may also account for inferior clinical outcomes. Therefore, no further evaluation of panitumumab has been performed.

While EXPAND and REAL3 evaluated EGFR inhibition in the first-line, COG assessed second-line gefitinib *versus* placebo in 449 unselected esophageal/GEJ adenocarcinoma or squamous cell carcinoma patients (55). Once again, COG failed to achieve its primary endpoint. Gefitinib demonstrated a modest mPFS benefit *versus* placebo (1.57 *vs* 1.17 months, HR 0.80, 95% CI 0.66–0.96) without an overall survival benefit (3.73 *vs* 3.67 months, HR 0.90, 95% CI 0.74–1.09) in unselected patients. However, 3% of gefitinib-treated patients achieved a partial response—lasting up to 7.33 months. TRANSCOG evaluated molecular correlations in tissue from patients enrolled in COG, including mutations in *EGFR, PIK3CA, BRAF*, and *KRAS* as well as copy number gain (38/292 patients) or *EGFR* amplification by FISH (21/292 patients) (56). While no differences were seen when stratifying by gene mutation status, patients with *EGFR* FISH+ tumors (either copy-number gain or amplification) derived a mPFS benefit (HR 0.42, 95% CI 0.22–0.81, p = 0.01) but not a mOS benefit (HR 0.57, 95% CI 0.30–1.06, p = 0.08) from gefitinib *versus* placebo in multivariate analysis. Most notably, the 21/292 (7.1%) patients with *EGFR*-amplified tumors achieved both a PFS and OS benefit with the addition of gefitinib, with a mPFS of 1.87 *vs* 0.97 months (HR 0.29, 95% CI 0.10–0.83, p = 0.021) and mOS 4.17 *vs* 1.70 months (HR 0.21, 95% CI 0.07–0.64, p = 0.006). Thus, the degree of *EGFR* amplification appears to predict EGFR inhibitor activity in mEGC.

Based upon these findings, a small cohort of patients with *EGFR-*amplified mEGC was treated with EGFR inhibitors. Patients received first-line FOLFOX+Abt-806, second-line FOLFIRI+cetuximab, or third or greater line cetuximab monotherapy. In this heterogeneous, though selected, population, 57% of patients achieved an objective response rate (ORR) with a mPFS of 10 months—including 14 months in a patient receiving cetuximab monotherapy (16, 59). Though the cohort was small and heterogeneous, these findings are consistent with the phase III subset analyses from EXPAND and TRANS-COG. As seen in ERBB2-targeting trials, this study also demonstrated resistance mechanisms to EGFR-directed therapies—namely selection of *RAS*, *PIK3CA*, and *ERBB2* altered and non-*EGFR*-amplified clones. Thus, tumor heterogeneity adds yet another layer of complexity to patient selection for targeted therapies in mEGC.

## Novel Approaches Targeting EGFR

Receptor tyrosine kinase blockade prolongs progression-free and even overall survival in many populations, but is limited by intratumoral heterogeneity and upregulation/activation of redundant or downstream signaling. Outside of mEGC, newer investigational agents attempt to deliver targeted cytotoxic payloads or prime an immune-mediated response.

Phase 1 data for MRG003, a fully human anti-EGFR IgG1 antibody conjugated to monomethyl auristatin E stabilized disease in an EGFR-expressing esophageal cancer patient that remains on treatment at 12 weeks (57). A similar phase I dose-escalation study of ABBV-321 (serclutamab talirine), an antibody-drug conjugate combining a humanized immunoglobulin G1 anti-EGFR antibody conjugated to a pyrrolobenzodiazepine dimer, remains underway in EGFR-over-expressing patients (NCT03234712) (60). One novel approach, EDV-D682, contains a bacterially derived EDV nanocell loaded with PNU-159682, a cytotoxic agent, and then coated with an EGFR antibody. In a recent phase 1 pancreatic cancer trial, EDV-D682 achieved radiographic disease control in 8/9 patients, including response in 4/5 evaluable patients, at 4 months. A phase 2 study is currently enrolling patients (ACTRN12619000385145) (61).

Another recent approach utilizes bispecific antibodies that recognize two distinct epitopes. One such agent, amivantamab, is a fully human anti-EGFR and c-MET-targeting antibody that demonstrated a 36% objective response rate and 10 month median response duration in patients with non-small cell lung cancers (NSCLC) harboring *EGFR* exon 20 insertions (62). The intent of this agent is to co-inhibit MET, which is a common resistance mechanism in EGFR-targeted NSCLC.

Rather than injecting antibodies, another EGFR-targeting approach undergoing investigation in pancreatic cancer collects autologous lymphocytes and expands them *in vitro* in the presence of OKT3 (anti-CD3) and cetuximab (anti-EGFR) in order to generate bispecific antibody armed T cells (BATs), which are then infused. Of “evaluable” patients in this seven patient phase 1/2 study, the median overall survival was 31 months despite lack of objective radiographic responses, with suggestion of an innate immune response, and better than expected responses to subsequent chemotherapy (63). A second line phase IB study is underway (NCT04137536) in order to confirm these findings.

In a similar manner, a phase 1 study of EGFR-targeted chimeric antigen receptor T-cells (CAR-T) was conducted in China, which demonstrated objective response in 2/11 and disease control in 7/11 patients, though survival data was immature at the time of publication (64). In addition to numerous ongoing EGFR CAR-T trials in China, pediatric evaluation of a second-generation agent targeting both 4-1BB and EGFR is underway in the United States (NCT03618381).

## Future Directions

Although all three phase III trials evaluating EGFR inhibition in mEGC failed to achieve their primary endpoints, EGFR biomarker analysis suggests that as in lung and colon cancers, EGFR inhibition has a significant role in a properly selected population. Retrospective analysis of targeting EGFR in *EGFR*-amplified mEGC patients by tissue and/or circulating tumor DNA next generation sequencing (8, 13), as well as prospective treatment with EGFR inhibitors, suggest that this may represent the ideal population for EGFR inhibition in future EGFR-targeting mEGC studies (16). However, investigators will need to ensure adequate definitions for positivity, as well as pre-specified stratification for baseline resistance mechanisms. Novel compounds including anti-EGFR antibody drug conjugates, bispecific antibodies, and cellular therapies may have a role in overcoming resistance mechanisms. Despite these limitations, six percent of the over one million EGC patients diagnosed each year represents a large patient population needing effective therapies, and so EGFR-targeted therapies merit re-evaluation.

## Author Contributions

All authors contributed to the article and approved the submitted version.

## Conflict of Interest

SM has received research support from Genentech and travel expenses from Merck and Bayer. YJ has received research funding from Boehringer Ingelheim, Bayer, Genentech/Roche, Bristol-Myers Squibb, Eli Lilly, and Merck and served on advisory boards for Merck Serono, Bristol-Myers Squibb, Eli Lilly, Pfizer, Bayer, Imugene, Merck, Daiichi-Sankyo, and AstraZeneca.

The remaining author declares that the research was conducted in the absence of any commercial or financial relationships that could be construed as a potential conflict of interest.
